# Comparison of the thermal and mechanical properties of concrete incorporating microencapsulated and macro-encapsulated phase change materials

**DOI:** 10.1038/s41598-026-49211-w

**Published:** 2026-04-17

**Authors:** Ziming Mao, Jingchao Li, Zhonglin Zhang, Yuhui Wu, Shaoshi Zhu, Jian Shang

**Affiliations:** 1 College of Water Conservancy and Hydropower Engineering, Hohai University, Nanjing, 210098 China; 2 Heilongjiang Province Water Resources Investment Group Co., Ltd, Harbin, 150040 China

**Keywords:** Phase change materials, Cooling-heating cycles, Microencapsulated phase change materials, Porous aggregates, Thermal and mechanical performance, Engineering, Materials science

## Abstract

The incorporation of phase change material (PCM) into concrete represents a promising approach to regulating internal temperatures and delaying the freezing process in cold regions. Currently, there are two primary methodologies for incorporating PCM into concrete: macro-encapsulated phase change aggregates (MPCAs), which are fabricated by incorporating PCM into porous carriers via vacuum adsorption, and microencapsulated phase change materials (MPCMs), which utilize a physical or chemical shell to enclose the PCM core. However, comparative studies evaluating the comprehensive performance of phase change concrete (PCC) integrated via these two encapsulation routes remain scarce. This study employed two types of MPCAs and two types of MPCMs to prepare PCCs, and evaluated the encapsulation performance of the aggregates for PCM as well as their thermal energy storage properties. Furthermore, the thermal regulation capacity and mechanical performance of the resulting PCCs were tested, alongside a systematic assessment of their transient thermal responses during cooling-heating cycles. Experimental results demonstrate that all prepared PCCs significantly delay temperature fluctuations, with a maximum delay freezing time of 3.41 h in pore water and a temperature reduction of 3.57 °C compared to ordinary concrete, effectively buffering severe internal temperature drops. Regarding the comparison of the two methodologies, although MPCMs exhibit superior encapsulation efficiency and thermal properties, their inherent mechanical fragility limits the 28d compressive strength of PCCs to only 12.3 and 8.8 MPa, failing to meet the requirements for load-bearing structures in cold regions. In contrast, MPCA-based PCCs achieve a balance between thermal regulation and structural performance, maintaining robust 28d compressive strengths of 31.3 and 28.5 MPa. This ensures the basic functional integrity of the PCC while demonstrating promising thermal regulation potential.

**Supplementary Information:**

The online version contains supplementary material available at 10.1038/s41598-026-49211-w.

## Introduction

In cold and high-altitude regions, concrete structures are exposed to frequent freeze–thaw cycles (FTCs), posing severe challenges to their long-term durability^1–3^. FTCs induce the initiation and propagation of internal cracks in concrete, leading to strength degradation and a consequent reduction in overall service performance^4^. To enhance the freeze–thaw resistance of concrete in low-temperature environments, mitigating pore water freezing fundamentally requires regulating the internal temperature field of the concrete. In this context, phase change material (PCM) have been demonstrated to serve as functional components in concrete^5–7^. Owing to their ability to absorb and release latent heat during phase transitions, PCM can mitigate temperature fluctuations induced by FTCs, thereby enhancing the freeze–thaw resistance of concrete structures. Consequently, PCM have gradually emerged as an important research direction in studies on the durability of concrete in cold regions.

The application of PCM in concrete is generally classified into high-temperature^8,9^ and low-temperature PCM^10,11^ according to their phase change temperature ranges. High-temperature PCM have been extensively investigated and applied in building thermal regulation and temperature control of mass concrete. In contrast, this study focuses on low-temperature PCM with the potential to mitigate freeze–thaw damage. Sakulich et al.^12^ were among the first to incorporate low-temperature PCM into cementitious systems using fine lightweight aggregates, and their work laid the foundation for subsequent related research. Paswan et al.^13^ further confirmed through differential scanning calorimetry (DSC) that, compared with conventional concrete, the incorporation of low-temperature PCM significantly lowers the freezing point of pore water, thereby delaying or even preventing internal FTCs. Yeon^14^ investigated the thermal performance of concrete incorporating graded PCM and examined the effect of covering thickness. The results indicated that graded PCMs can significantly broaden the phase change temperature range and improve the freeze–thaw resistance, while the covering thickness has a limited effect on the overall thermal insulation performance. Deb et al.^15^ applied phase change concrete in outdoor snow-melting experiments. The results showed that the snow-melting and temperature-regulating performance of the PCM is highly dependent on the ambient temperature prior to snowfall, and when the environmental temperature is significantly below their phase change point, the temperature-regulating effect is markedly limited. Overall, existing studies have demonstrated that low-temperature PCM can slow the rate of internal temperature variation in concrete through latent heat absorption and release. This allows concrete to remain within a relatively moderate temperature range before ambient temperatures rise or fall again, thereby reducing the risk of damage caused by rapid freezing or thawing of pore water.

The incorporation of PCM into concrete can generally be classified into two main approaches. One approach involves the direct use of microencapsulated phase change materials (MPCMs)^16–20^, while the other relies on porous aggregates to absorb and encapsulate PCM, serving as carrier media^21^. MPCMs offer good encapsulation integrity and dispersion, effectively preventing PCM leakage during mixing and enabling relatively uniform temperature regulation at the microscale^22^. However, the encapsulating shell materials often reduce the overall thermal conductivity and generally possess limited mechanical strength, which may adversely affect the mechanical performance of concrete^23,24^. In contrast, incorporating PCMs through porous aggregates allows a larger amount of phase change medium to be accommodated, thereby markedly enhancing the thermal regulation and energy storage capacity of concrete^25^. Meanwhile, the relatively rigid aggregate skeleton helps maintain satisfactory mechanical performance^26–29^. Nevertheless, this approach still faces challenges, including limited adsorption efficiency^30,31^, potential leakage of the PCM, and the inherently lower strength of lightweight aggregates^32–35^. Consequently, the temperature-regulation mechanisms, durability performance, and overall influence on the macroscopic properties of concrete differ substantially between these two PCM incorporation strategies. Clarifying their respective performance characteristics and applicable conditions is therefore essential for advancing the practical implementation of PCM-modified concrete in cold-region engineering.

Currently, comprehensive comparative studies focusing on the thermal, mechanical, and freeze-thaw performance of concrete incorporating PCMs through the two incorporation strategies remain limited. To address this gap, the present study develops phase change concrete by replacing conventional coarse and fine aggregates with two types of macro-encapsulated phase change aggregates (PCAs) and two types of microencapsulated PCMs (MPCMs), respectively. At the aggregate scale, the leakage behavior of PCAs and MPCMs during continuous thermal cycling was evaluated, and their thermophysical properties were characterized using differential scanning calorimetry (DSC). Subsequently, at the concrete scale, the influence of incorporation methods and replacement ratios on the temperature-regulating capacity and compressive strength was systematically investigated. Furthermore, the potential freeze-thaw durability was assessed to elucidate the performance characteristics of different PCM integration strategies, providing a theoretical foundation for the practical implementation of PCM-modified concrete in cold-region engineering.

## Experiment program

### Raw materials

#### Phase change material

**Fig. 1:**
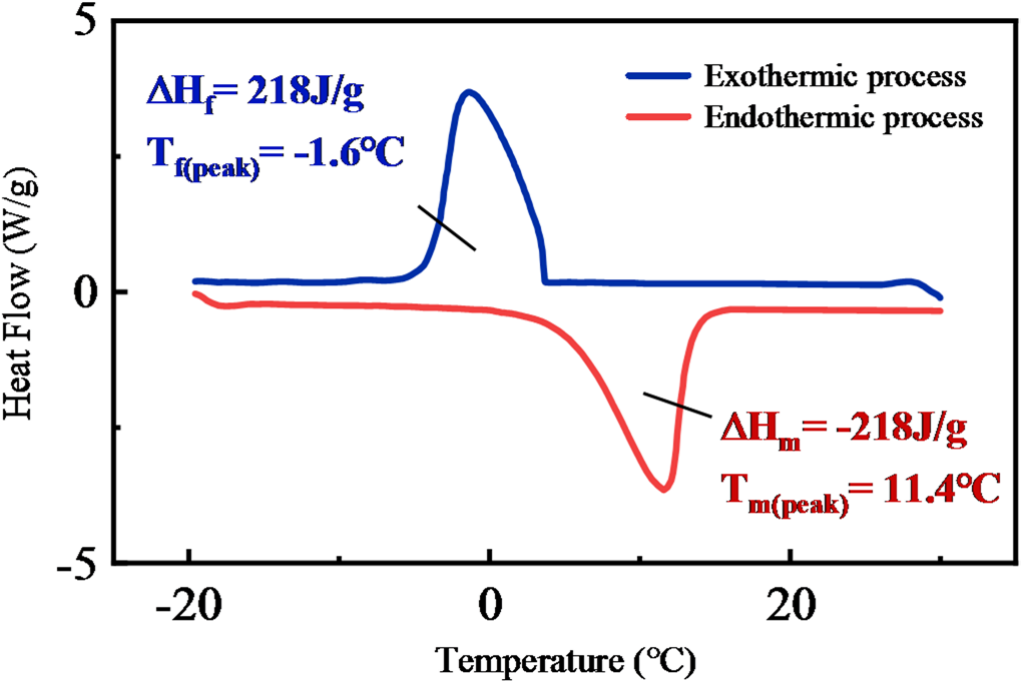
DSC testing results of the PCM.

To ensure consistency of variables, paraffin was selected as the sole PCM in this study, owing to its stable latent heat during phase transition and reliable chemical properties^36,37^. The selected paraffin exhibits a phase transition temperature of approximately 2 °C during the cooling process and about 5 °C during the heating process. Figure 1 presents the DSC thermal analysis results of the PCM.

#### Aggregates

**Fig. 2:**
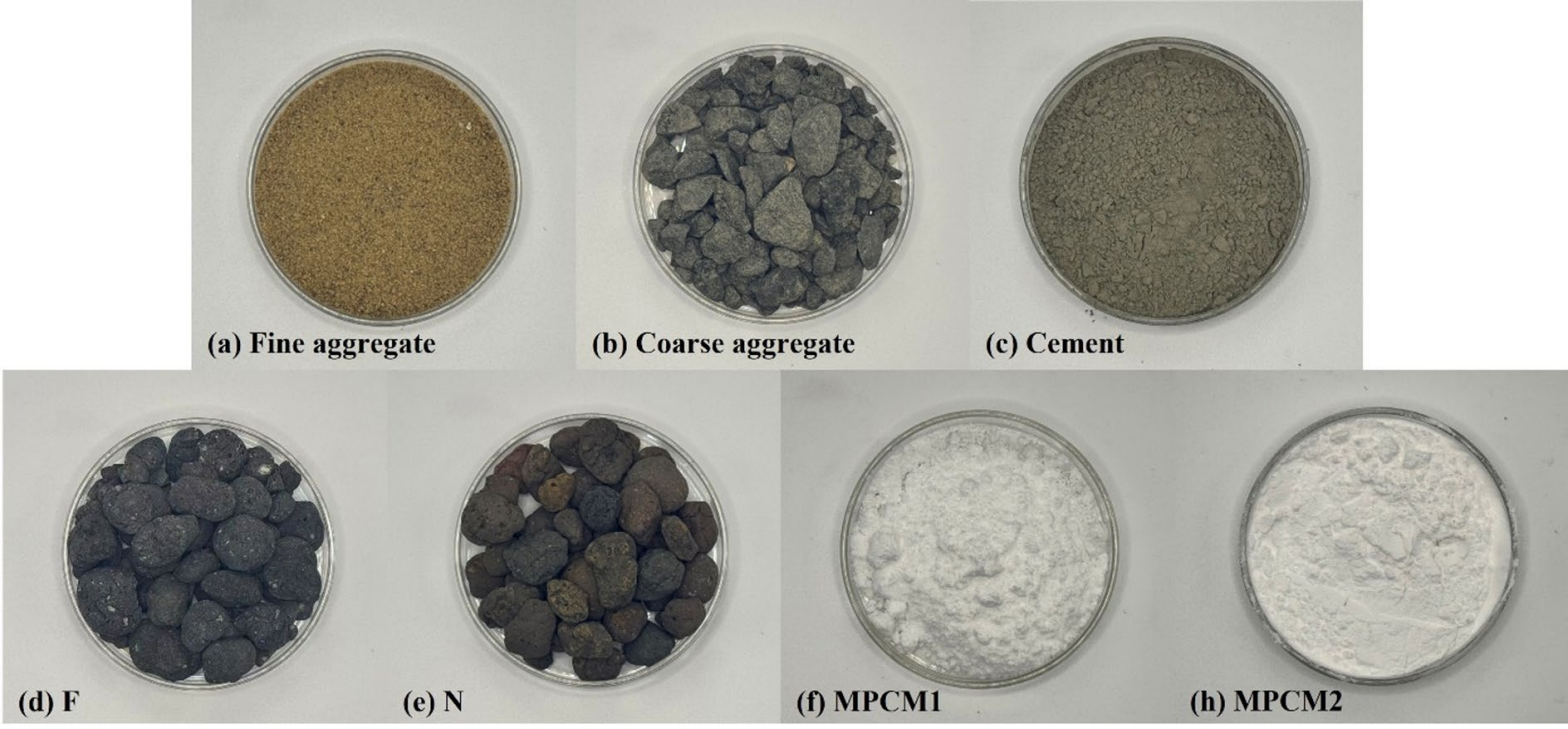
Morphologies of the raw materials used in this study.

Basalt crushed stone with a particle size range of 5–20 mm was used as the coarse aggregate, owing to its high strength and good stability, making it suitable for concrete preparation under freeze–thaw conditions. Standard river sand with a uniform particle-size distribution was selected as the fine aggregate to ensure adequate workability and mechanical performance of the mixtures. Figure 2 presents the morphologies of the raw materials used in this study.

#### Cementitious materials

The cementitious materials consisted of ordinary Portland cement (PO 42.5) and Class I fly ash. The cement was used to provide the matrix strength, while the fly ash served as a mineral admixture to improve the workability of the fresh mixture and promote the development of later-age properties^38,39^.

### Preparation method

#### Macro-encapsulated phase change materials

**Fig. 3:**
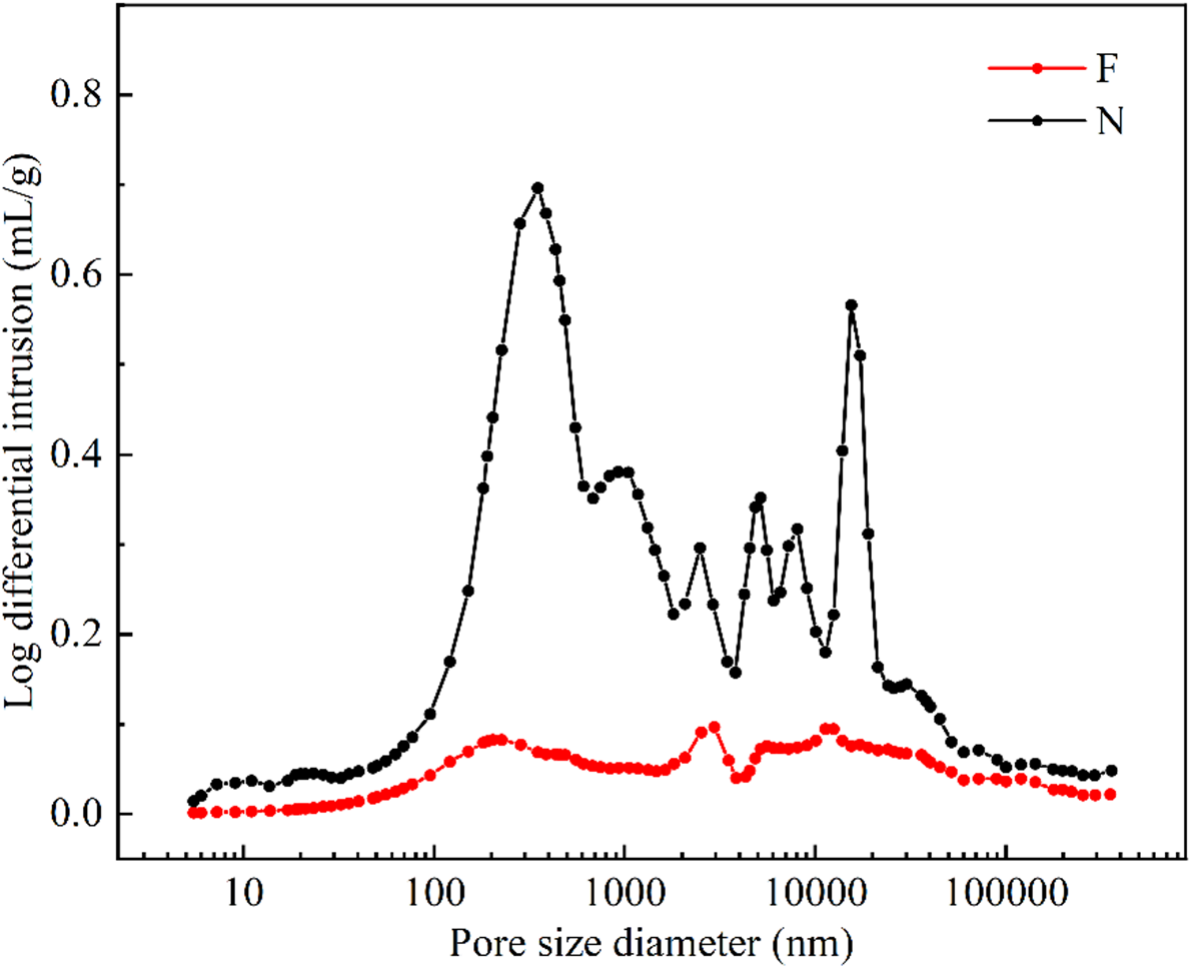
Pore size distributions of the porous aggregates F and N.

Two types of porous aggregates with significantly different pore structures, labeled as F and N, were selected as carriers for macro-encapsulated phase change aggregates (MPCAs), with fly ash and clay as the primary raw materials. The porous aggregates were prepared through a re-sintering process, during which an appropriate amount of pore-forming gas was added to create a stable porous structure at high temperature. The resulting aggregates exhibited notable differences in porosity and pore size characteristics, making them suitable for subsequent PCM encapsulation and performance evaluation. The basic physical properties of F and N are presented in Table 1, and their pore size distributions are illustrated in Fig. 3.

**Table 1:** Basic physical properties of the porous aggregates F and N.

Number	Apparent density (kg/m^2^)	Bulk density (kg/m^2^)	Porosity (%)
F	1000	500	54.63
N	1900	900	35.73

**Fig. 4:**
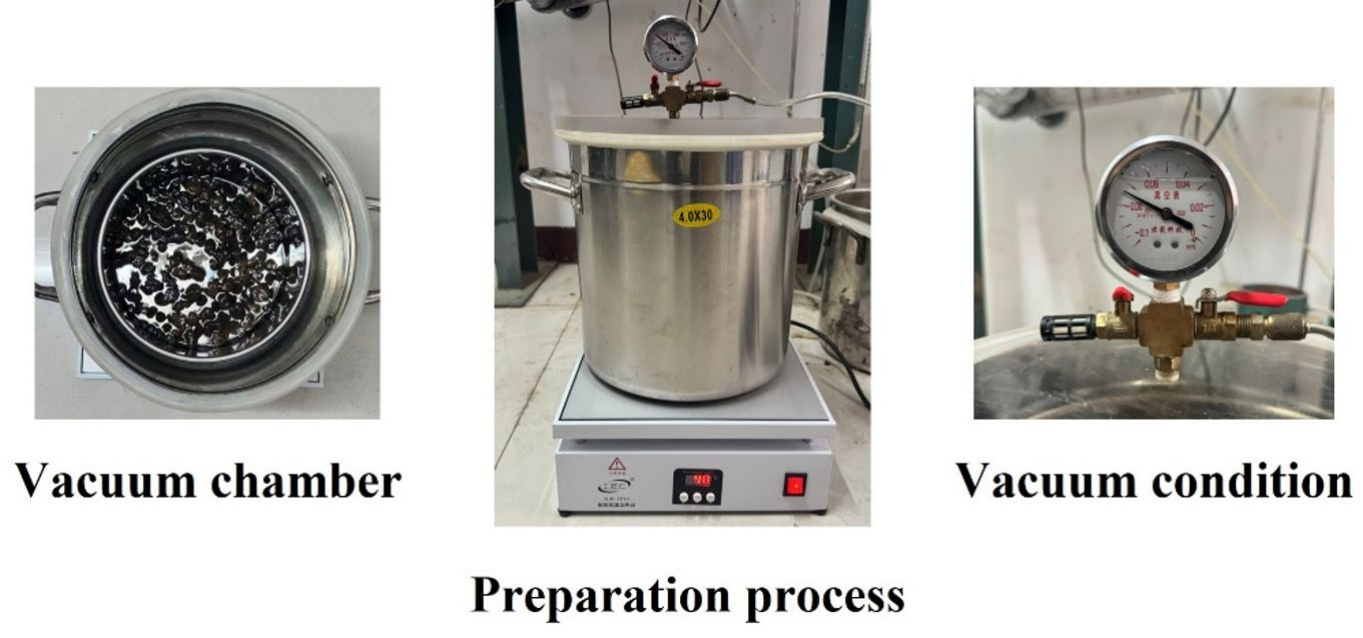
Schematic illustration of the preparation procedure for MPCAs.

MPCAs were prepared using a vacuum impregnation method. The preparation procedure was as follows. First, the porous aggregates were thoroughly rinsed and then oven-dried at 105 °C for 12 h. The dried aggregates were subsequently placed in a vacuum chamber and maintained at a pressure of − 0.08 MPa to evacuate the air within the pore structure. While maintaining the vacuum condition, liquid paraffin was introduced until the liquid level exceeded the aggregate bed by 3 cm, depending on the adsorption capacity of the aggregates. The system was then heated to 55 °C and maintained for 12 h to ensure sufficient impregnation of the PCM. Figure 4 illustrates the preparation process of the MPCAs. The volumetric occupancy rates of PCM within the prepared PCM-F and PCM-N are determined to be 29.9% and 38.1%, respectively.

To prevent residual paraffin on the aggregate surface from adversely affecting cement hydration during concrete casting and curing, a surface treatment was conducted after impregnation^40,41^. The prepared phase change lightweight aggregates were wrapped with oil-absorbing paper and spread evenly on trays for 24 h, during which the aggregates were turned over every 2 h. Subsequently, the aggregates were rinsed with warm water at a temperature slightly above the phase transition temperature of paraffin for 5 min, until only a negligible amount of oily substance was observed in the discharged water. This surface treatment method has been demonstrated to be effective in previous studies^42^.

#### Microencapsulated phase change materials

The microcapsule shells, composed of organic melamine formaldehyde (MF) resin and inorganic silica (SiO₂), were synthesized via in situ polymerization and sol–gel methods, respectively. To prepare the MPCMs, a paraffin based core was first emulsified to form stable oil phase droplets. Subsequently, shell precursors were introduced to undergo surface polycondensation^43^ or controlled deposition^44^, forming a dense protective barrier. This encapsulation strategy ensures high thermal stability and structural integrity. Following curing, washing, and drying, the resulting MPCMs exhibited superior sealing performance and morphological stability, suitable for integration into the cementitious matrix. The characterization results indicate that the mass fraction of the active PCM core within the prepared MPCMs is 85%, demonstrating a high encapsulation efficiency. Furthermore, the particle size distribution analysis reveals a D90 value of 20 μm.

#### Preparation of phase change concretes

Phase change concrete (PCC) was prepared according to the designed mix proportions. Prior to mixing, the MPCAs were saturated with water to ensure a stable internal moisture condition, whereas the MPCMs were added directly to the mixture in a dry state. During mixing, the dry constituents were first blended thoroughly, followed by the addition of mixing water. The mixture was then mixed using a forced action mixer until a homogeneous consistency was achieved. After mixing, the fresh concrete was cast into molds and fully compacted on a vibrating table. The specimens were fabricated as 100 × 100 × 100 mm cubic samples and cured under standard conditions until the designated testing ages.

**Fig. 5:**
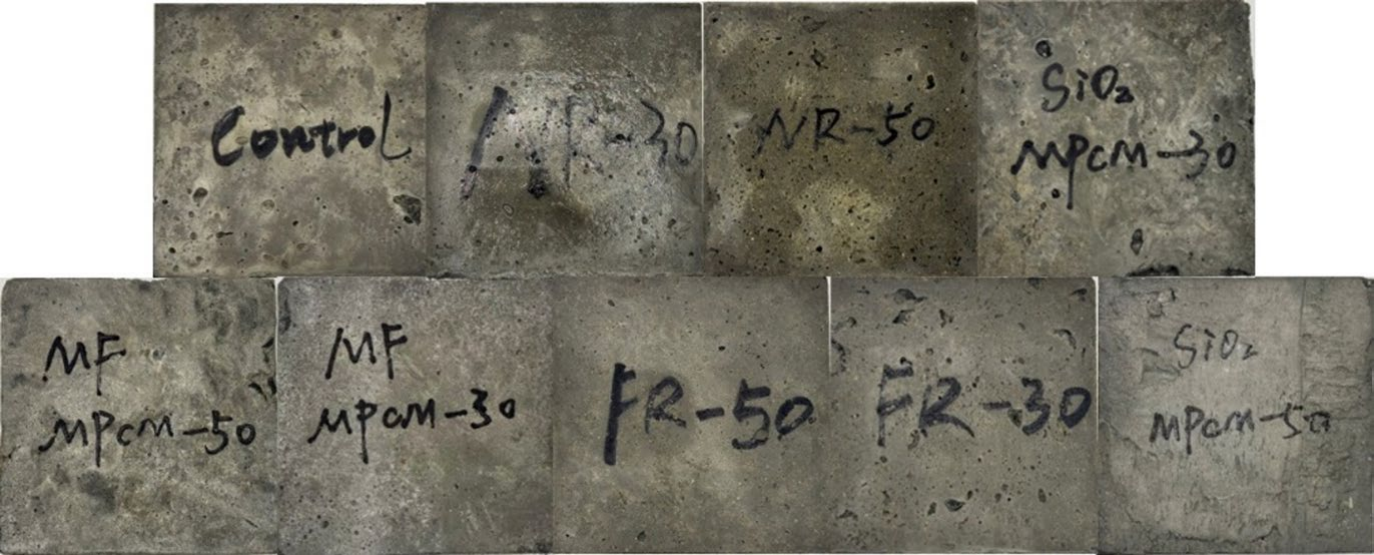
Appearance of the prepared concrete specimens.

A systematic labeling convention was adopted to identify the concrete mixtures. The control group is designated as C. For PCCs, groups incorporating MPCAs (types F and N) as coarse aggregate replacements are labeled as FR and NR, respectively. Groups utilizing MPCMs as fine aggregate replacements are denoted as M1 (SiO₂ shell) and M2 (MF shell). Based on prior empirical evidence^7,19,40^, the replacement rates of 50% and 30% can respectively ensure a conservative mechanical integrity and an essential thermal regulation performance for the PCCs. The suffixes “-50” and “-30” represent the volumetric replacement percentages of the aggregates. The detailed mix proportions of the prepared concretes are summarized in Table 2. Figure 5 presents the appearance of the prepared concrete specimens.

**Table 2:** Mix proportions of the prepared concretes.

Number	Water/kg	Concrete/kg	Sand/kg	Gravel/kg	MPCMs/kg	LWA-PCM/kg	Fly ash/kg	PCM content/kg
C	180	425	650	1061	0	0	98	0
NR-30	180	425	650	740	0	160	98	52
NR-50	180	425	650	530	0	265	98	87
FR-30	180	425	650	740	0	230	98	37
FR-50	180	425	650	530	0	377	98	63
M1-30	180	425	455	1061	80	0	98	68
M1-50	180	425	330	1061	130	0	98	113
M2-30	180	425	455	1061	70	0	98	60
M2-50	180	425	330	1061	116	0	98	100

### Experimental methods

#### Properties of phase change aggregates

To evaluate the thermal stability of the phase change aggregates, two types of MPCAs and the MPCMs were subjected to 20 FTCs over a temperature range from − 20 °C to + 20 °C. Qualitative filter paper was placed beneath the samples to absorb any PCM that might leak during the cycling process. After the FTCs, the leakage of the PCM from the aggregates was evaluated by observing the extent of PCM impregnation on the qualitative filter paper, and the impregnated area was recorded.

The thermal properties of the MPCAs and the MPCMs were characterized using DSC. A TA Instruments DSC-250 was employed to measure the phase change temperature and latent heat over a temperature range of − 30 to 20 °C at a heating and cooling rate of 5 °C/min. Prior to testing, the MPCAs were freeze-dried and ground into powders with particle sizes of 1–2 mm to ensure measurement consistency.

#### Thermal and mechanical properties of phase change concrete

**Fig. 6:**
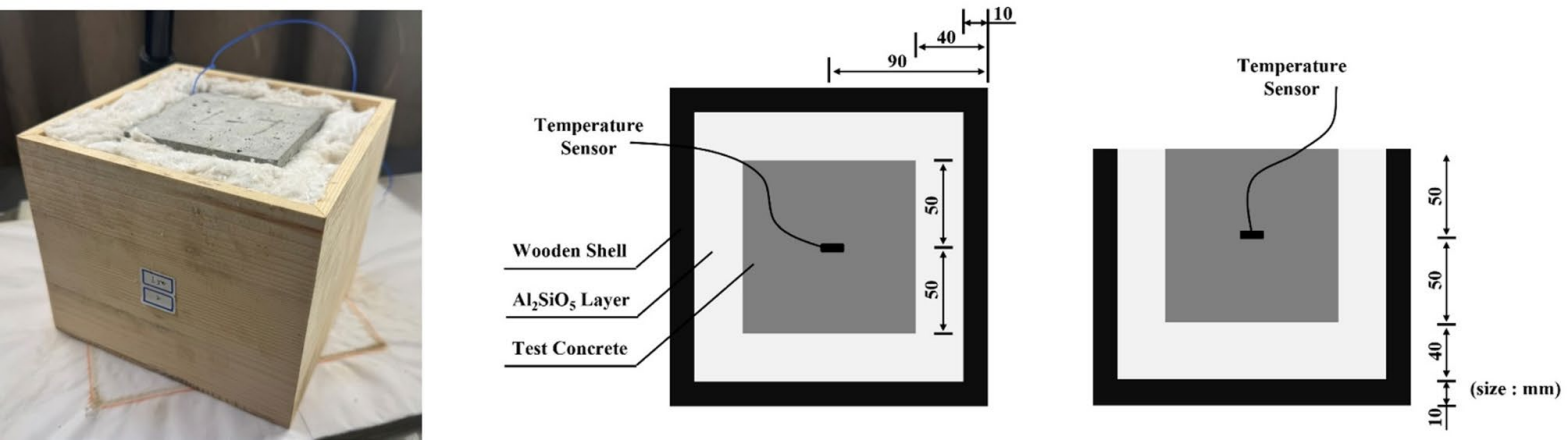
Schematic diagram of the single sided freezing simulation chamber for concrete.

To evaluate the thermal insulation and temperature regulation performance of PCC within a single FTC, a self-developed low-temperature testing apparatus was employed to investigate its thermal response behavior. Figure 6 illustrates the schematic diagram of the single sided freezing simulation chamber for concrete. During the test, the prepared concrete specimens were placed in an open-top cubic wooden box, with insulation layers of 30–40 mm thickness installed on the side walls and bottom to simulate single-sided freezing conditions encountered in real service environments. Since the horizontal dimensions of the concrete chamber were much larger than the freezing depth, heat transfer was assumed to occur only in the depth direction, and one-dimensional heat conduction was considered. To record the internal temperature evolution of each specimen, a K-type thermocouple was embedded at the geometric center of the concrete cube for continuous temperature monitoring.

The compressive strength of concrete specimens at curing ages of 3, 7, and 28 days was tested. The load was applied continuously and uniformly at a loading rate of 3 kN/s until specimen failure. At least three parallel specimens were tested for each group, and the average value was taken as the final result to ensure the reliability and repeatability of the test results.

The microstructures of four types of phase change concrete after compression failure were observed using an SU3500 scanning electron microscope (SEM). After completion of the mechanical tests, representative specimens were selected and crushed to expose fresh fracture surfaces, followed by drying treatment. SEM observations were conducted to examine the pore structure of the cement matrix, the characteristics of the interfacial transition zone, and the distribution and integrity of phase change aggregates within the cementitious matrix. A comparative analysis was then performed to evaluate the effects of different PCM incorporation methods on the microstructure and damage characteristics of PCC.

## Results and discussion

### Freeze–thaw stability of aggregates

**Fig. 7:**
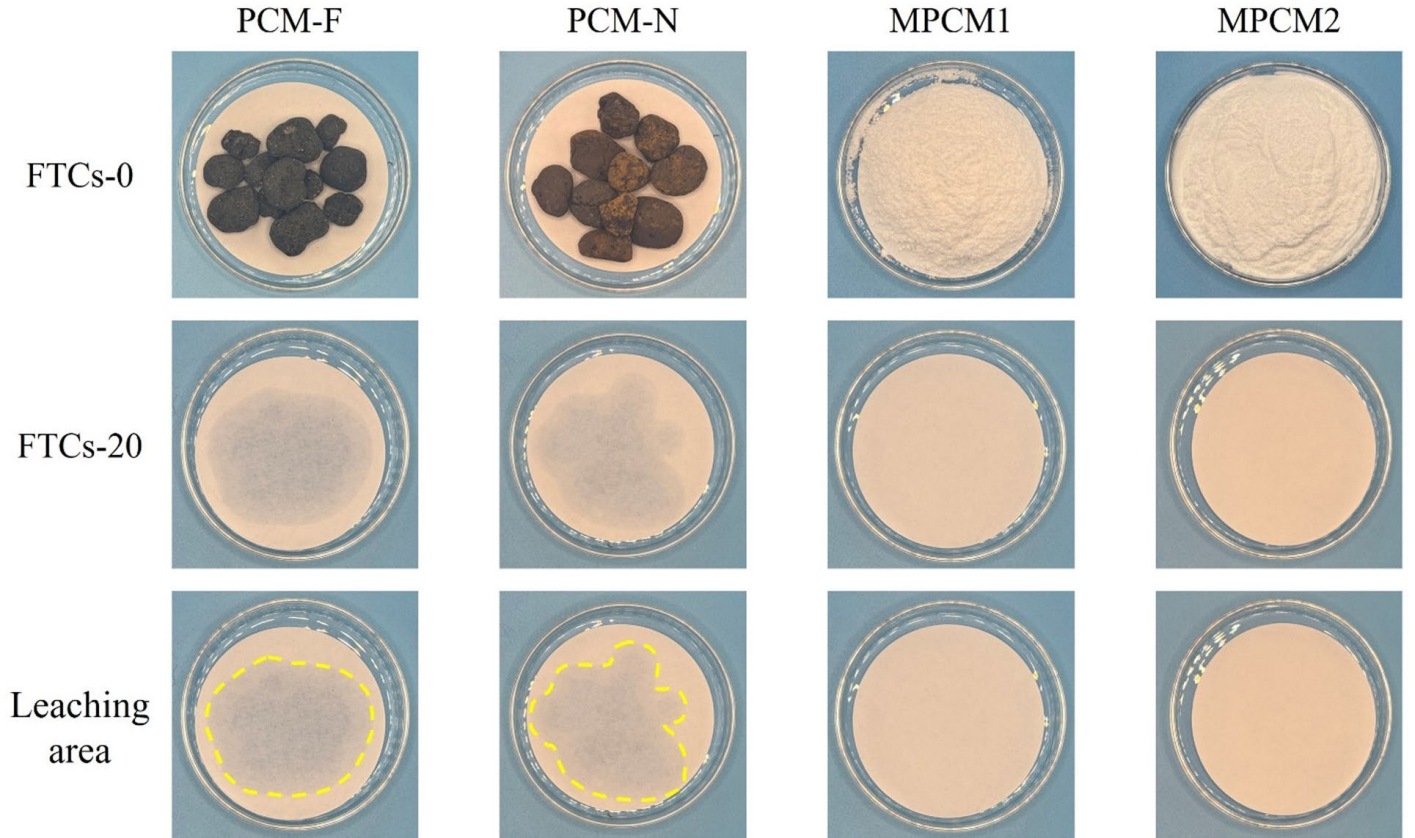
Leakage behavior of phase change aggregates after 20 freeze-thaw cycles.

Figure 7 shows the leakage behavior of PCM from the phase change aggregates after 20 FTCs. As shown in the figure, the macro-encapsulated phase change aggregates exhibit significant leakage after 20 FTCs. The staining area on the filter paper almost entirely overlaps with the original placement of the aggregates, indicating an omnidirectional and non-directional outflow of PCM. This extensive staining reflects the structural degradation of the internal pore network within the aggregates induced by FTCs. According to the MIP results, both aggregates F and N possess a considerable number of macropores, which exert a relatively weak confinement effect on the PCM. Under the action of continuous thermal cycling, the capillary pressure that originally confined the PCM within these pores gradually diminishes, compromising its retention capacity and consequently leading to the leakage of the liquid phase. The MPCMs exhibited no significant leakage after undergoing the same number of FTCs. This indicates that the polymer shells of the microcapsules possess excellent structural toughness and sealing integrity, which can effectively resist the thermal stresses generated by severe temperature fluctuations.

### Thermal properties of aggregates

**Fig. 8:**
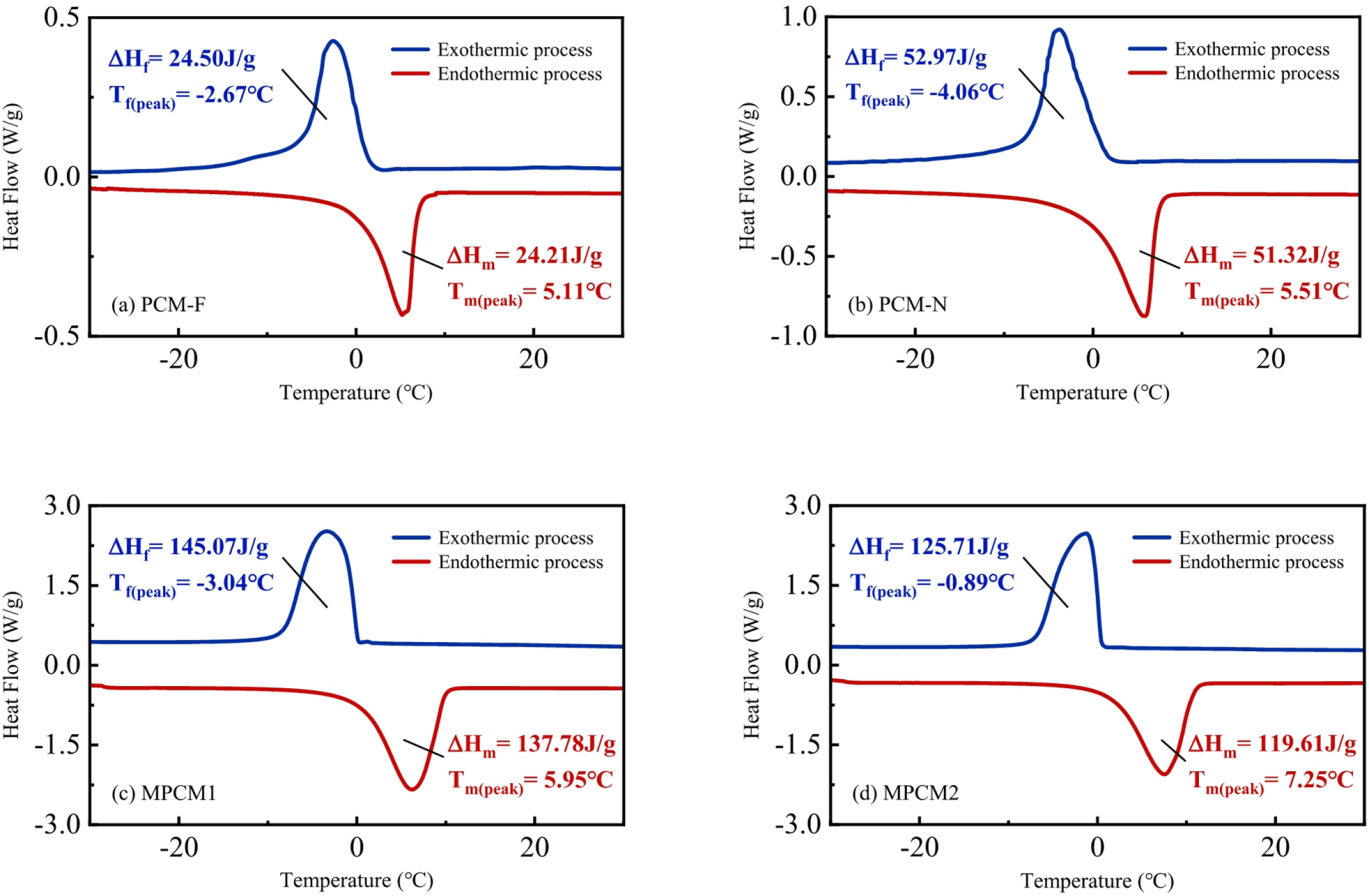
DSC test results of the prepared phase change aggregates.

Figure 8; Table 3 presents the DSC thermal characteristics of MPCAs and MPCMs. As indicated by the DSC curves, all types of phase change aggregates exhibit distinct exothermic peaks, the peak ranges of which are closely related to the amount of PCM stored within the aggregates. In general, a higher exothermic peak intensity indicates a larger amount of effective PCM participating in the phase transition, thereby contributing more significantly to temperature regulation. Under the same mass conditions, MPCMs exhibit higher latent heat, which is attributed to the much lower mass ratio of the capsule shell to the PCM compared with that of MPCAs. Among the same type of phase change aggregates, PCM-N shows a higher latent heat than PCM-F, because the porous aggregate N has a higher porosity and can therefore store a larger amount of PCM. In addition, the latent heat of MPCM1 is slightly higher than that of MPCM2.

**Table 3:** Detailed DSC parameters of the phase change aggregates.

Number	Cooling stage				Heating stage			
	Onset point (°C)	Peak point (°C)	End point (°C)	Latent heat (J/g)	Onset point (°C)	Peak point (°C)	End point (°C)	Latent heat (J/g)
PCM-F	1.59	− 2.67	− 14.09	24.5	− 3.21	5.11	9.05	24.50
PCM-N	1.76	− 4.06	− 10.55	52.97	− 3.09	5.51	8.49	51.32
MPCM1	0.18	− 3.04	− 8.05	145.07	0.54	5.95	9.87	137.78
MPCM2	0.53	− 0.89	− 6.73	125.71	1.76	7.25	10.70	119.61

Further analysis shows that the phase change peaks of MPCMs are relatively broader, whereas the MPCAs exhibit a wider phase change temperature range. This indicates that, under the combined effects of encapsulation materials and pore confinement, the same PCM may exhibit different phase change behaviors. The relatively broad yet concentrated exothermic peak of MPCMs suggests a more uniform particle size distribution, resulting in a relatively stable phase change temperature. In contrast, the porous aggregates possess a much wider pore size distribution, leading to significant differences in the confinement conditions of the stored PCM, which is manifested as a broad distribution of phase change temperatures within the same macro-encapsulated aggregate.

In terms of the phase change sequence, during the cooling process, the phase change onset temperatures of all types of phase change aggregates are concentrated between 0 and 2 °C, which ensures that the PCMs start releasing heat before the pore water begins to freeze. The phase change peak temperatures are located between − 4 and 0 °C, corresponding to the stage of concentrated heat release, slightly higher than the freezing point of pore water in the concrete. This indicates that the PCM can effectively and fully perform their thermal regulation function during the cooling stage.

### Temperature response during freeze–thaw cycles

**Fig. 9:**
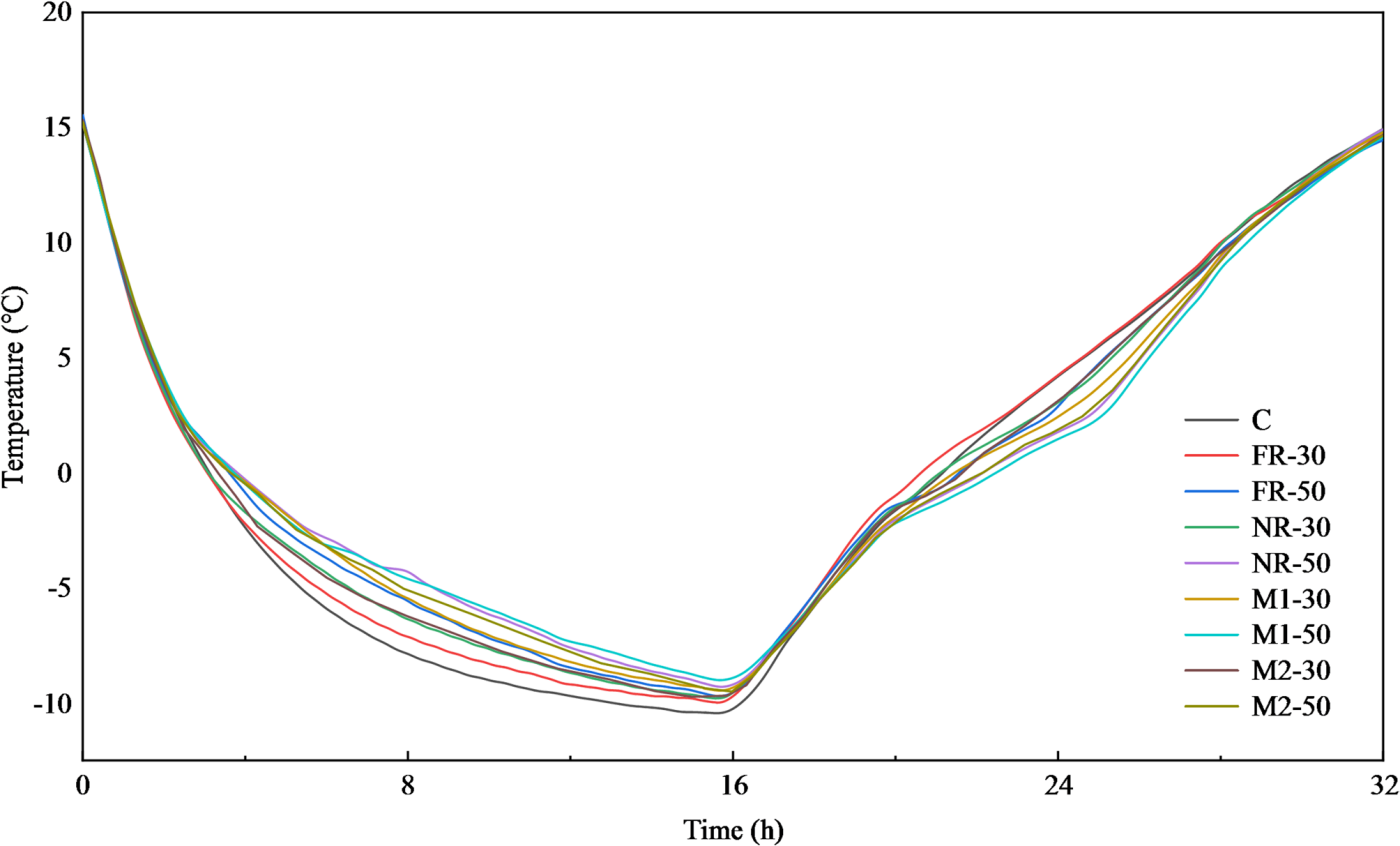
Temperature responses of concrete specimens during a single cooling-heating thermal cycle.

Figure 9 and Table 4 illustrates the temperature responses of all specimens during a single cooling-heating thermal cycle. During the initial cooling stage (before the concrete temperature decreased to 2 °C), the cooling rates of all PCCs were essentially the same as that of the control concrete. This indicates that when the ambient temperature is outside the phase change temperature range of the PCM, the incorporation of phase change aggregates does not impair the inherent thermal conductivity or insulation performance of the concrete. When the concrete temperature decreased to approximately 2 °C, the cooling rate of the PCCs was significantly reduced, whereas no comparable temperature inflection point was observed in the control concrete. This behavior can be attributed to the PCM approaching the onset of phase transition, during which part of the PCM transformed from the liquid to the solid state and released latent heat. The released heat effectively counteracted the temperature decrease within the specimens over a short period, thereby markedly slowing the cooling rate of the PCCs. Meanwhile, differences in the cooling rate reduction were observed among the PCCs with different aggregate replacement ratios. In general, PCCs with higher PCM contents exhibited a more pronounced temperature-delay effect. Among them, the NR-50, M1-50, and M2-50 concretes showed the most significant cooling-delay performance.

**Table 4:** Delayed freezing time and maximum temperature difference of PCCs compared with C.

Number	Cooling stage		Heating stage	
	Delayed freezing time (h)	Max. temperature difference (°C)	Delayed melting time (h)	Max. temperature difference (°C)
FR-30	0.44	0.76	− 0.53	0.35
FR-50	2.18	2.34	0.40	1.38
NR-30	1.45	1.57	0.02	1.15
NR-50	3.32	3.57	1.13	2.65
M1-30	2.18	2.71	0.35	1.81
M1-50	3.41	3.31	1.31	3.11
M2-30	1.26	1.66	0.48	1.10
M2-50	3.35	2.93	1.30	2.51

When the concrete temperature dropped to the range of -2 °C to -6 °C, both the control concrete (C) and the PCCs exhibited a significant deceleration in cooling rate, with some samples even showing a transient temperature rebound. This phenomenon is primarily attributed to the supercooling effect of the pore water within the concrete matrix. The pore water remains in a metastable liquid state below its theoretical freezing point until the degree of supercooling triggers spontaneous nucleation^43,45^. Subsequently, the rapid release of latent heat of crystallization during the cooling process compensates for the environmental heat loss, leading to a distinct rise in the internal temperature of the specimens. Taking − 5 °C as the concentrated freezing temperature of pore water, M1-50, M2-50, FR-50, and NR-50 successfully delayed the time to reach this freezing threshold by 3.41, 3.35, 2.18, and 3.32 h, respectively. Overall, this freezing delay capacity is generally positively correlated with the actual PCM content within the different PCCs. However, the MPCAs demonstrate a superior actual efficiency in exerting their thermal regulation effects. As the cooling process progressed, the degree of temperature divergence among the various specimen groups became increasingly significant. Among all groups, M1-50, M2-50, and NR-50 exhibited the most outstanding thermal lag capacity, which was specifically reflected in the substantial temperature differential between their internal temperature curves and that of C. This demonstrates that the high content PCM effectively delayed the declining trend of the internal concrete temperature by releasing latent heat, thereby enhancing the thermal stability of the material. The data regarding the delayed freezing time and maximum temperature difference of PCCs compared with C are recorded in Table 4.

During the heating stage, the PCCs similarly demonstrates the ability to retard the temperature rise rate, which is attributed to the substantial latent heat absorption by the PCM during its solid-to-liquid transition. However, its effectiveness in delaying the phase change of pore water is significantly inferior to that observed during the cooling stage. This discrepancy primarily arises because the phase change temperature of most PCMs is higher than the melting point of water. Consequently, along the heating path, the endothermic action of the PCM occurs after the thawing of pore water, preventing it from effectively buffering the ice-to-water transition in the same manner as the pre-emptive heat release during the cooling stage.

However, it is crucial to acknowledge the long-term limitations of the MPCAs. Although PCCs incorporating MPCAs exhibit promising performance in short-term thermal and mechanical evaluations, the relatively high leakage rate associated with these porous aggregates poses a significant hidden threat to the long-term durability of the concrete structure. Under continuous FTCs, the unconfined liquid PCM is prone to gradually exude from the aggregate matrix and migrate through the surrounding cementitious network. This continuous loss of PCM will inevitably deplete the effective PCM content within the concrete, thereby substantially weakening its long-term thermal regulation capability over its service life.

### Mechanical properties

**Fig. 10:**
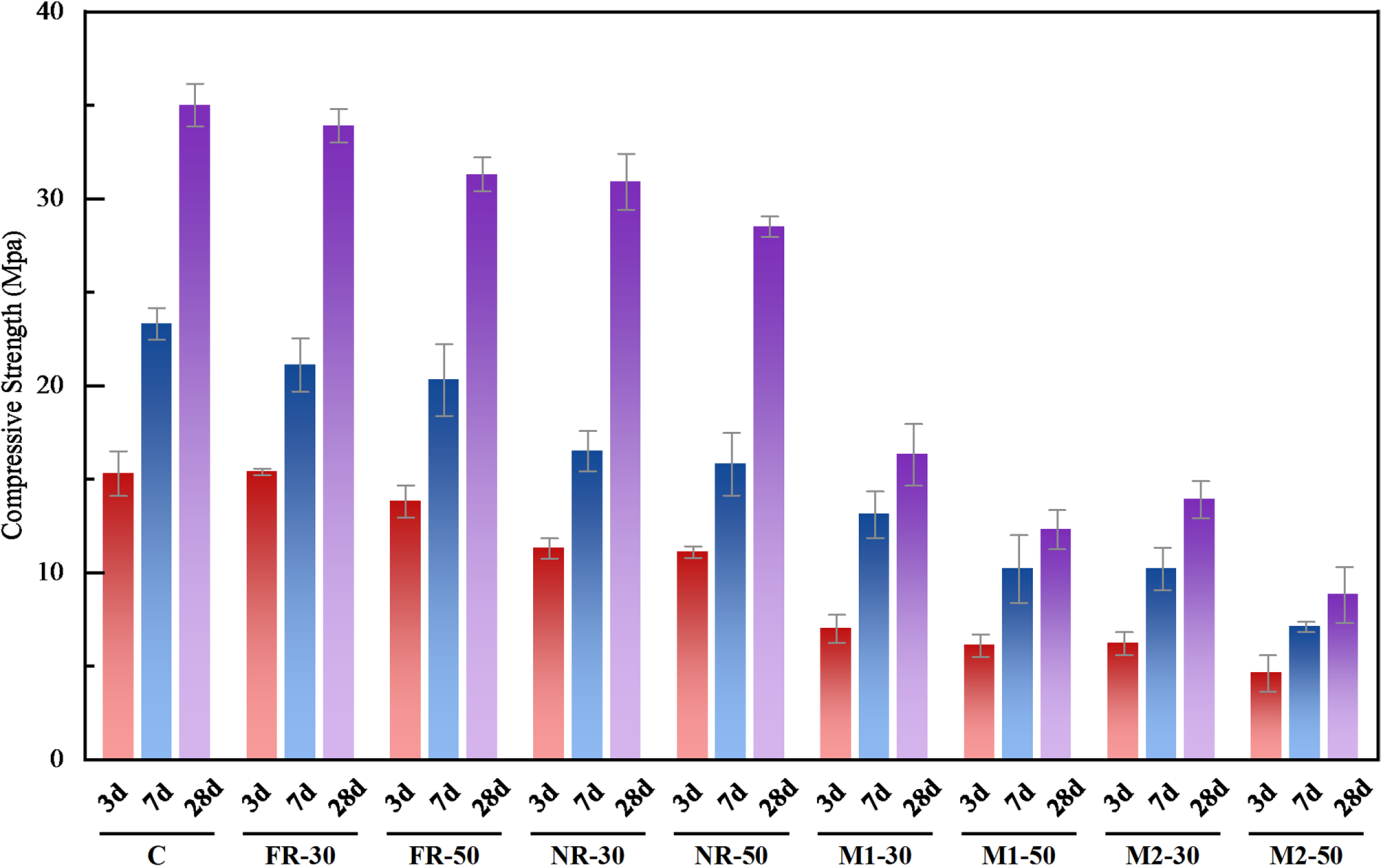
Compressive strength development of different concrete mixtures.

**Fig. 11:**
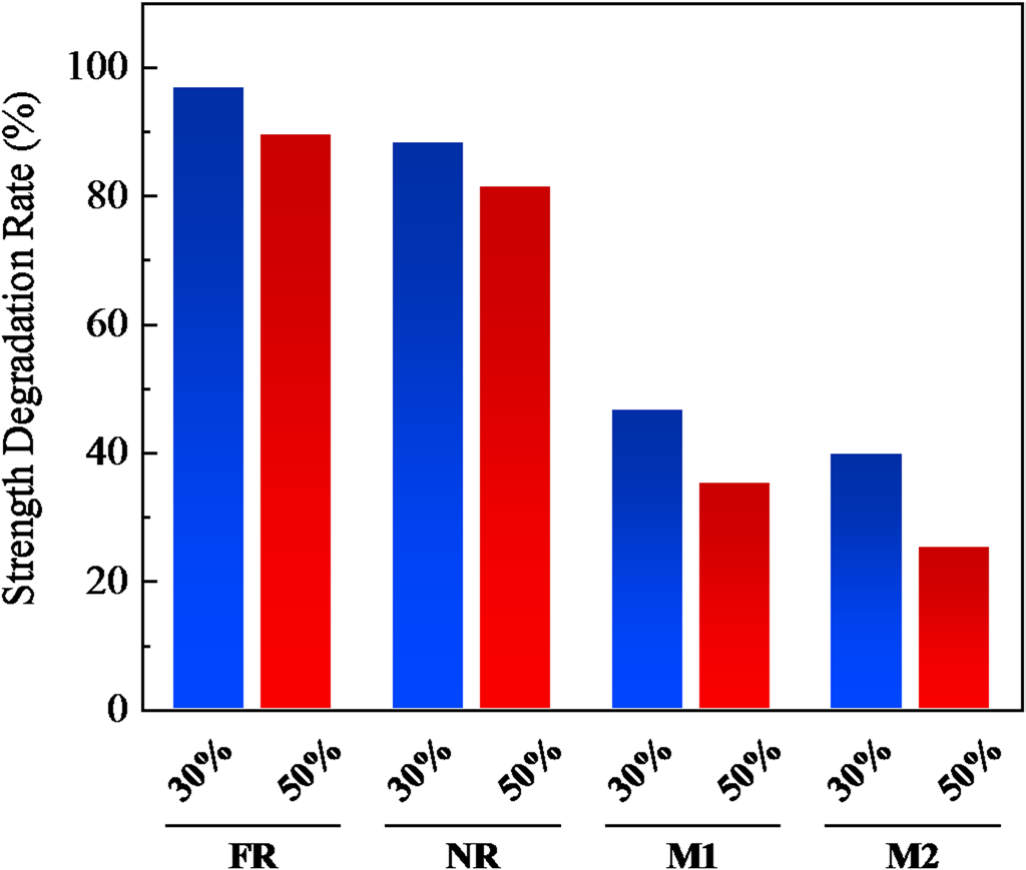
Strength retention rate of PCCs relative to C.

Figures 10 and 11 illustrates the compressive strength development and strength retention ratios of different concrete mixtures. The 28d compressive strength of the control concrete is 35.0 MPa. After the incorporation of phase change aggregates, all PCCs exhibited varying degrees of strength reduction, with the loss being more pronounced in the PCCs containing MPCMs.

When MPCM1 was used to replace 30% and 50% of the fine aggregate, the 28d compressive strengths of the PCCs were 16.3 MPa and 12.3 MPa, corresponding to strength retention ratios of only 47% and 35% of the control mixture, respectively. Similarly, when MPCM2 replaced 30% and 50% of the fine aggregate, the 28d compressive strengths decreased to 13.9 MPa and 8.8 MPa, with strength retention ratios of 40% and 25%, respectively. These strength reductions are mainly attributed to the relatively low mechanical properties of the microcapsule shell materials and the introduction of a large number of weak interfaces within the cement matrix, which deteriorate the interfacial transition zone and consequently weaken the overall load-bearing capacity of the PCCs.

PCCs incorporating MPCAs exhibit a comparatively favorable mechanical performance. When PCM-F was used to replace 30% and 50% of the coarse aggregate, the 28d compressive strengths of the PCCs were 33.9 MPa and 31.3 MPa, corresponding to strength retention ratios of 96% and 89% of the control concrete, respectively. When PCM-N replaced 30% and 50% of the coarse aggregate, the 28d compressive strengths decreased to 30.9 MPa and 28.5 MPa, with corresponding strength retention ratios of 88% and 80%. The porous aggregates exhibit sufficient mechanical strength to contribute to the load-bearing framework of the concrete. Their particle strength and stiffness are significantly higher than those of MPCMs, allowing them to effectively participate in the load-transfer system after replacing conventional aggregates. Moreover, the rough surface texture and dense pore structure of the porous aggregates promote mechanical interlocking and interfacial bonding with the cement paste, enabling the PCCs to maintain satisfactory mechanical performance while retaining its temperature-regulating capability.

Among the same category of PCCs, M1 exhibits relatively higher mechanical performance, which is closely associated with the differences in the shell materials used for the two MPCMs. When SiO_2_ is employed as the shell material, its higher stiffness and mechanical strength, together with better compatibility with cementitious materials, promote the formation of a denser and more stable interfacial structure, thereby partially mitigating the strength loss induced by the incorporation of PCM. In contrast, when MF is used as the shell material, its inherently lower mechanical strength and the formation of an organic–inorganic interface with the cement paste result in weaker interfacial bonding. Under mechanical loading, such interfaces are more susceptible to crack initiation and propagation, which ultimately leads to a further reduction in the overall compressive strength of the concrete. For MPCAs, FR exhibit higher compressive strength than NR, mainly due to the denser load-bearing skeleton formed by the lower porosity of FR. Nevertheless, from an engineering perspective, the compressive strength of NR remains within an acceptable range and is sufficient to meet basic mechanical performance requirements.

From the perspective of compressive strength development, the strength growth rate of PCCs incorporating MPCMs is significantly restricted in the later stages. Regarding the underlying mechanism, this severe degradation is attributed to the synergistic combination of restricted hydration, increased porosity, and the inherent weakness of the microcapsules. Specifically, the massive specific surface area of MPCMs adsorbs substantial free water, which not only lowers the effective water-to-cement ratio to restrict localized hydration but also severely degrades workability, leading to massive entrapped air and agglomeration defects during casting. Furthermore, these MPCMs act as soft inclusions with a low elastic modulus; their extensive incorporation occupies the growth space for hydration products and creates a physical barrier that disrupts the continuity of the calcium silicate hydrate (C-S-H) gel network. Consequently, the contribution of late-stage hydration to the structural skeleton’s strength is markedly diminished^5^. In contrast, MPCAs exhibit a different effect: the free water stored within their pores can exert an internal curing effect. By continuously releasing moisture into the surrounding matrix, it promotes secondary hydration of the cement, thereby partially offsetting the negative impact of the PCM on the mechanical properties^12^.

**Fig. 12:**
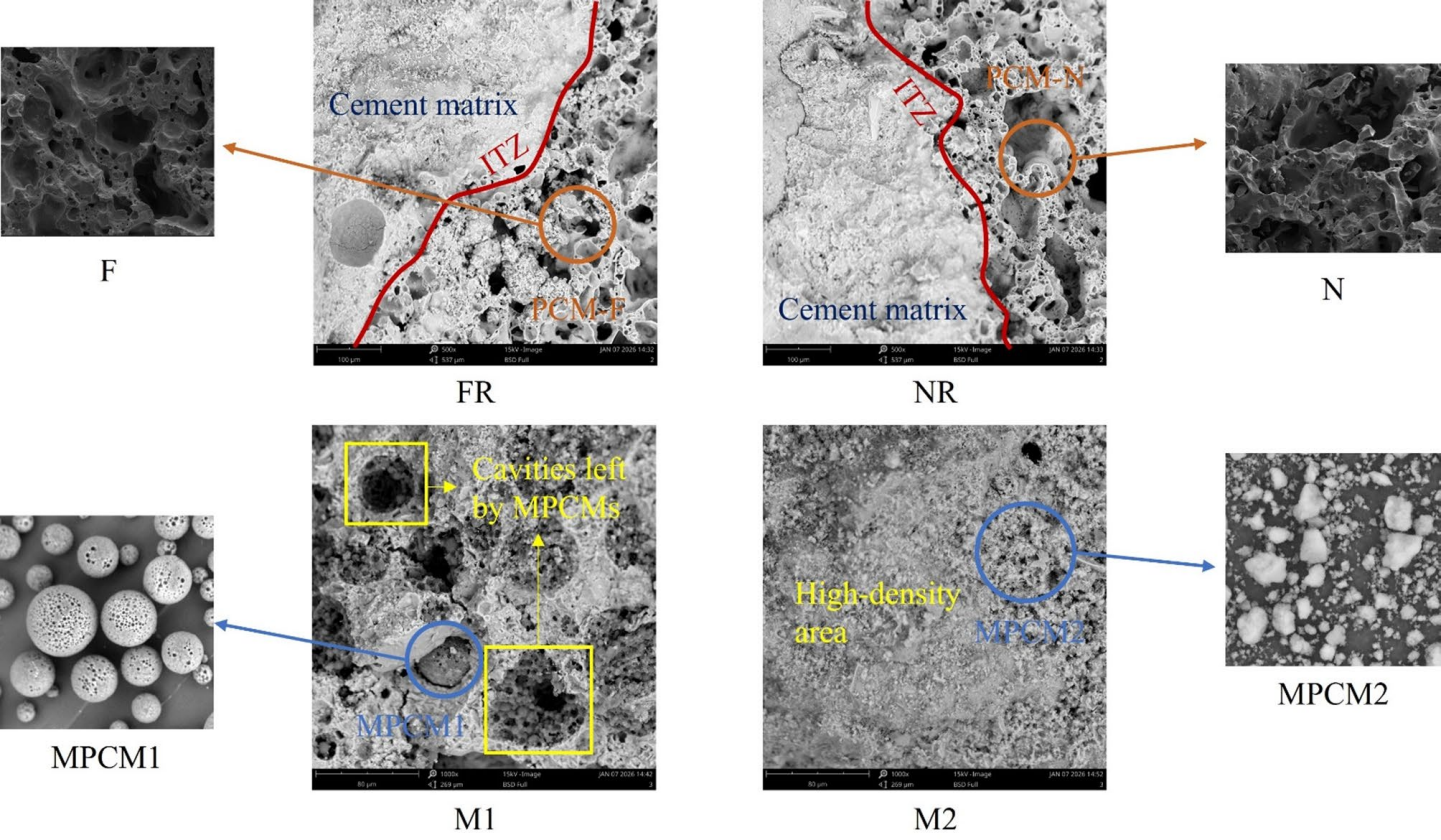
SEM images of the PCCs after compressive failure.

Figure 12 presents the SEM images of the PCCs after compressive failure. Observations reveal that the rough surface of the porous aggregates is encapsulated by an abundance of granular hydration products, manifesting a robust mechanical interlocking effect within the interfacial transition zone (ITZ). Despite the fracture of aggregates during the failure process, the contact interface between the aggregates and the cement paste remains remarkably tight, with no visible voids or interfacial micro-cracks. This potent interfacial bonding, complemented by the inherent structural strength of the porous aggregates, collectively sustains the mechanical framework of the concrete^46^.

Regarding the PCCs incorporating MPCMs, MPCM1 exhibits a regular spherical morphology under microscopic observation, with dense micropores distributed on its shell surface. When embedded within the cement matrix, a distinct spherical outline is visible. However, the smooth outer surface of MPCM1 results in a prominent gap at the interface with the cement matrix. This microstructural discontinuity suggests a lack of effective physical anchoring and chemical bonding between the MPCM1 and the cement paste, causing the interfacial transition zone (ITZ) to act as a structural weak point. MPCM2 exhibits a fragmented, powder-like morphology under microscopic observation, possessing almost no inherent load-bearing capacity^20,24^. When distributed throughout the cement matrix, these powder-like particles fail to establish effective mechanical interlocking with the concrete; instead, they significantly disrupt the structural continuity of the cement paste. This loose distribution creates extensive stress concentration zones and mechanical weak planes within the matrix, leading to premature structural instability under compression.

## Conclusion

This study comparatively investigated the physical properties of two types of MPCAs and two types of MPCMs. A comprehensive assessment was conducted on the thermal and mechanical performance of the resulting PCCs. Furthermore, the thermal responses of the PCCs during cooling-heating cycles were systematically evaluated, providing insights into their thermal regulation potential for cold-region engineering. The following conclusions are drawn based on the comparative investigation of MPCAs and MPCMs characteristics and the comprehensive evaluation of the resulting PCCs:

(1) From the aggregate perspective, MPCMs exhibit significantly higher encapsulation stability than MPCAs under FTCs. Due to the protection of dense shells, MPCMs show negligible PCM leakage without mechanical compromise, whereas MPCAs are prone to the loss of surface-adhered PCM. In terms of thermal capacity, MPCMs possess a higher latent heat storage density per unit mass owing to their superior core-to-shell ratio. However, while the phase change range of MPCMs is relatively narrow due to their uniform particle size, MPCAs offer a broader temperature interval for heat absorption and release. This is attributed to their multi-scale pore structures and heterogeneous pore size distributions, which provide greater flexibility for thermal regulation in varying environments.

(2) Both incorporation methods endowed the PCCs with excellent thermal regulation performance. Influenced by the content of PCM, the thermal buffering capacity followed the order of M1 > NR > M2 > FR. During the cooling phase, the PCCs significantly retarded the cooling rate, delaying the pore water freezing point by 3.41, 3.32, 3.35, and 2.18 h, respectively, compared to the control concrete, with maximum temperature gradients of 3.31, 3.57, 2.93, and 2.34 °C. However, during the heating phase, the protective effect against ice melting was less pronounced, as the phase change temperature of the PCM was slightly higher than the melting point of ice. Furthermore, a threshold effect was observed in the dosage level, indicating that a critical amount of PCM is required to fully activate its temperature-regulating potential.

(3) Significant disparities in mechanical performance were observed between PCCs prepared with the two incorporation methods. At a 50% replacement level, the 28d compressive strengths of FR and NR were 31.3 and 28.5 MPa, representing minor reductions of only 11% and 20% compared to C. In contrast, M1 and M2 exhibited drastic strength losses of 65% and 75%, with values dropping to 12.3 and 8.8 MPa, respectively. These results demonstrate that MPCAs possess a far superior load-bearing skeletal capacity than MPCMs. Microscopic analysis further confirms that the MPCAs are well embedded within the cement matrix, forming a high-integrity interfacial bond. Moreover, the internal skeletal structure of MPCAs remains dense even after concrete crushing. Conversely, distinct separation gaps exist at the MPCM-cement interface, which not only hinders load transfer but also compromises the structural integrity of the concrete matrix.

In conclusion, while MPCMs exhibit a significant advantage in PCM containment by effectively preventing leakage, they act as mechanical defects within the concrete matrix. The excessive reduction in initial strength caused by PCM incorporation renders them inadequate to meet the basic load-bearing requirements for structural applications in cold regions. Conversely, MPCAs demonstrate superior structural compatibility by serving as a load-bearing skeleton. In practical engineering, PCCs integrated with MPCAs demonstrate promising short-term thermal regulation potential. hey achieve a feasible balance between thermal regulation and acceptable mechanical properties based on current short-term evaluations, fulfilling the dual requirements of energy saving and structural safety. Furthermore, addressing the inherently low thermal conductivity of organic PCMs remains a critical challenge for maximizing the thermal storage efficiency of PCCs. Future investigations should prioritize advanced enhancement strategies, such as incorporating highly thermally conductive additives or engineering optimized encapsulation shells, to accelerate the transient thermal response^16–18^. Meanwhile, the long-term freeze-thaw durability of both types of PCCs warrants further experimental validation to ensure the feasibility of their practical engineering applications.

## Supplementary Information

Below is the link to the electronic supplementary material.

Supplementary Material 1

## Data Availability

The datasets during the current study available from the corresponding author on reasonable request.
