# Supplementary material for: Comparison of the thermal and mechanical properties of concrete incorporating microencapsulated and macro-encapsulated phase change materials
Source: Sci Rep. 2026 Apr 17;16:17788. doi: 10.1038/s41598-026-49211-w (PMC13247205; doi:10.1038/s41598-026-49211-w)
Supplement: Supplementary file 1 — Supplementary Material 1 [file 41598_2026_49211_MOESM1_ESM.docx]

**---------------------------------------------------Title Page------------------------------------------------**

# Article title:

Comparison of the thermal and mechanical properties of concrete incorporating microencapsulated and macro-encapsulated phase change materials

# Author names:

**1st author name:** Ziming Mao^1^

**E-mail:** [mzm@hhu.edu.cn](mailto:mzm@hhu.edu.cn)

**2nd author name:** Jingchao Li^2^

**E-mail:** 5396679@163.com

**3rd author name:** Zhonglin Zhang^2^

**E-mail:** zhangzhonglin_11@163.com

**4th author name:** Yuhui Wu^1^

**E-mail:** [wyh001@hhu.edu.cn](mailto:wyh001@hhu.edu.cn)

**5th author name:** Shaoshi Zhu^2^

**E-mail:** [15326651526@163.com](mailto:15326651526@163.com)

**6th author name:** Jian Shang^2, *^

**E-mail:** 18545821110@163.com

# Affiliations and addresses:

^1^ College of Water Conservancy and Hydropower Engineering, Hohai University, Nanjing 210098, China.

^2^ Heilongjiang Province Water Resources Investment Group Co., Ltd., Harbin 150040, China.

# * Corresponding author:

**Name:** Jian Shang

**E-mail:** 18545821110@163.com
